# Suppression of methane uptake by precipitation pulses and long-term nitrogen addition in a semi-arid meadow steppe in northeast China

**DOI:** 10.3389/fpls.2022.1071511

**Published:** 2023-01-16

**Authors:** Weifeng Gao, Xu Yang, Yicong Zhang, Tianhang Zhao, Baoku Shi, Tianxue Yang, Jianying Ma, Wanling Xu, Yining Wu, Wei Sun

**Affiliations:** ^1^ Institute of Grassland Science, Key Laboratory of Vegetation Ecology of the Ministry of Education, Jilin Songnen Grassland Ecosystem National Observation and Research Station, Northeast Normal University, Changchun, Jilin, China; ^2^ State Environmental Protection Key Laboratory of Wetland Ecology and Vegetation Restoration, Northeast Normal University, Changchun, Jilin, China; ^3^ Key Laboratory of Geographical Processes and Ecological Security in Changbai Mountains, Ministry of Education, School of Geographical Sciences, Northeast Normal University, Changchun, Jilin, China; ^4^ College of Geography and Ocean Sciences, Yanbian University, Hunchun, China; ^5^ College of Wildlife and Protected Area, Northeast Forestry University, Harbin, China

**Keywords:** precipitation pulse, long-term N addition, methane, suppression effect, meadow steppe

## Abstract

In the context of global change, the frequency of precipitation pulses is expected to decrease while nitrogen (N) addition is expected to increase, which will have a crucial effect on soil C cycling processes as well as methane (CH_4_) fluxes. The interactive effects of precipitation pulses and N addition on ecosystem CH_4_ fluxes, however, remain largely unknown in grassland. In this study, a series of precipitation pulses (0, 5, 10, 20, and 50 mm) and long-term N addition (0 and 10 g N m^-2^ yr^-1^, 10 years) was simulated to investigate their effects on CH_4_ fluxes in a semi-arid grassland. The results showed that large precipitation pulses (10 mm, 20 mm, and 50 mm) had a negative pulsing effect on CH_4_ fluxes and relatively decreased the peak CH_4_ fluxes by 203-362% compared with 0 mm precipitation pulse. The large precipitation pulses significantly inhibited CH_4_ absorption and decreased the cumulative CH_4_ fluxes by 68-88%, but small precipitation pulses (5 mm) did not significantly alter it. For the first time, we found that precipitation pulse size increased cumulative CH_4_ fluxes quadratically in both control and N addition treatments. The increased soil moisture caused by precipitation pulses inhibited CH_4_ absorption by suppressing CH_4_ uptake and promoting CH_4_ release. Nitrogen addition significantly decreased the absorption of CH_4_ by increasing NH_4_^+^-N content and NO_3_^–^-N content and increased the production of CH_4_ by increasing aboveground biomass, ultimately suppressing CH_4_ uptake. Surprisingly, precipitation pulses and N addition did not interact to affect CH_4_ uptake because precipitation pulses and N addition had an offset effect on pH and affected CH_4_ fluxes through different pathways. In summary, precipitation pulses and N addition were able to suppress the absorption of CH_4_ from the atmosphere by soil, reducing the CH_4_ sink capacity of grassland ecosystems.

## Introduction

Methane (CH_4_) is the second-largest greenhouse gas in the atmosphere, with a relative global warming potential over a 100-year horizon (GWP-100) of 27.9 times that of carbon dioxide (IPCC, 2021). Surprisingly, atmospheric CH_4_ concentrations increased as high as 1866.3 ± 3.3 ppb in 2019, 156% greater than pre-industrial levels (729.2 ± 9.4 ppb) and the largest over the past 800,000 (IPCC, 2021). These increased CH_4_ concentrations can raise the global surface temperature by impacting radiation processes (Milich, 1999; Boucher et al., 2009). It is estimated that atmospheric CH_4_ contributes to approximately 20% of global radiative forcing and is an essential contributor to global warming (Dalal and Allen, 2008; IPCC, 2021). Exchanges of CH_4_ between the atmosphere and soil involve complex biological processes that depend on the comprehensive performance of CH_4_ production by methanogens and consumption by methanotrophs (Le Mer and Roger, 2001; Freitag et al., 2010). More specifically, CH_4_ is generated through methanogenesis by methanogens under anaerobic conditions (Conrad et al., 2007; Juottonen, 2020), while it is oxidized and consumed under aerobic conditions by methanotrophs, a type of microbe that uses CH_4_ as their unique carbon (C) source (Freitag et al., 2010; Judd et al., 2016). Natural ecosystems contribute significantly to CH_4_ fluxes into the atmosphere and act as a source of CH_4_ (Dalal and Allen, 2008; Houweling et al., 2017; IPCC, 2021). Grassland is, however, recognized as a major natural sink of atmospheric CH_4_, consuming 3.03-3.73 Tg CH_4_ yr^-1^; this makes these environments crucial components in regulating the global CH_4_ budget and greenhouse effect as grassland plays an essential role in balancing atmospheric CH_4_ concentration (Zhuang et al., 2013; Yu et al., 2017). In the context of global climate change, the changes in precipitation pulses and N deposition significantly affect CH_4_ fluxes (Aronson and Helliker, 2010; Harms and Grimm, 2012; Petrakis et al., 2017; Deng et al., 2020). The interactive effects of precipitation pulses and N addition on CH_4_ fluxes, however, remain largely unknown in the grassland.

Since the 1870s, continued global warming has altered the global water cycles as well as precipitation regimes (Bichet et al., 2011; IPCC, 2021). The precipitation pulses and precipitation patterns have changed significantly (IPCC, 2021). Compared with precipitation pulses, scientists paid more attention to the effects of precipitation patterns on CH_4_ fluxes (Billings et al., 2000; Chen et al., 2013; Aronson et al., 2019; Yue et al., 2022). The response of CH_4_ fluxes to the precipitation pulses, however, is largely unknown. Precipitation pulses are an essential method of supplying supplementary water to the soil in natural terrestrial ecosystems, especially in arid and semi-arid regions (Noy-Meir, 1973). In the future, the occurrence of precipitation pulses is expected to decrease, while occurrences of heavy pulses are expected to increase on a global scale, altering soil biogeochemical cycling processes and ecosystem functions (Norton et al., 2008; Griffin-Nolan et al., 2021; IPCC, 2021). Ecologists have found that precipitation pulses cause an increase in soil water availability, ammonium nitrogen (NH_4_^+^-N), nitrate nitrogen (NO_3_^–^-N), dissolved organic carbon (DOC), and aboveground biomass (AGB) (Norton et al., 2008; Chen et al., 2009; Shen et al., 2015; Leitner et al., 2017; Zhao et al., 2017; Liu et al., 2019), decreases in soil temperature and soil O_2_ concentrations (Han et al., 2018; Niu et al., 2019), and shifts in redox conditions as well as the metabolic and community structures of soil microbes (Harms and Grimm, 2012; Wang et al., 2016; Petrakis et al., 2017). Precipitation pulses, therefore, could induce a pulse effect (also called the “Birch effect”) of greenhouse gas fluxes (Norton et al., 2008; Kim et al., 2012; Zhao et al., 2017). Although the pulse effects and driving mechanisms of precipitation pulses on carbon dioxide and nitrous oxide fluxes have been intensively studied, little is known about the response of CH_4_ fluxes to these changing precipitation pulses (Huxman et al., 2004; Norton et al., 2008; Chen et al., 2009; Kim et al., 2012). Previous studies have shown that small precipitation pulses do not significantly alter the CH_4_ fluxes in the forests or steppes examined because precipitation did not result in the substantial changes to soil water content required to affect CH_4_ production (Mariko et al., 2007; Ni et al., 2019). Higher precipitation pulses (31.8 mm and 200 mm) were able to stimulate an increase of up to 23,479% CH_4_ release in a temperate forested watershed and desert floodplain (Harms and Grimm, 2012; Petrakis et al., 2017). In contrast, more extreme precipitation pulses (203 mm and 208 mm) suppressed the absorption of CH_4_ in the grassland and even shifted the grassland ecosystem from a CH_4_ sink to a source (Zhao et al., 2017; Ren et al., 2019). This indicates that CH_4_ flux changes in response to precipitation pulses differ based on location, and much is still unknown about how different environments respond to precipitation pulses. How CH_4_ fluxes behave in response to a series of precipitation pulses also needs more study, especially examined in the context of long-term nitrogen addition.

Nitrogen (N), as an essential element, is the most limiting nutrient in arid and semi-arid grassland ecosystems (Wang et al., 2014a). Atmospheric N deposition in many parts of the world has substantially increased over the past decades (Galloway et al., 2004; Liu et al., 2013). The N enrichment on land surfaces can both alleviate N limitations and profoundly affect C cycling processes. Previous studies indicate that N addition could increase soil N availability, soil organic carbon, AGB, litter quality, and litter decomposition rates, decrease soil pH and cause acidification, and change the community structure and abundance of soil microbes and the CH_4_ release processes they mediated (Kruger and Frenzel, 2003; Treseder, 2008; Lu et al., 2014; Gong et al., 2015; Yang et al., 2017; Ji et al., 2019; Kong et al., 2019; Liu et al., 2021b). Various meta-analyses suggest that N addition could enhance the release of CH_4_ from the soil; in contrast, small amounts of N addition could stimulate CH_4_ uptake instead, while larger N addition tends to inhibit CH_4_ uptake from the atmosphere to the soil (Aronson and Helliker, 2010; Deng et al., 2020). The exact effect of long-term N addition on CH_4_ fluxes, therefore, is largely uncertain. Additionally, there may be complex interactions between N addition and precipitation pulses that affect CH_4_ fluxes. The CH_4_ fluxes response to the interaction effects on precipitation pulses and long-term N addition remains largely unknown.

In this study, the responses of CH_4_ fluxes to precipitation pulses of different sizes and long-term N addition were assessed in a semi-arid meadow steppe. The objectives were: (1) to assess the effects of precipitation pulses on the dynamic change and patterns on CH_4_ fluxes; (2) to examine the effects of long-term N addition on CH_4_ fluxes; and (3) to examine the interactive effects of precipitation pulses and N addition on CH_4_ fluxes. It was hypothesized that (1) the precipitation pulses would have a negative pulse effect on CH_4_ fluxes and shift the ecosystem from a CH_4_ sink to a source; (2) precipitation pulses and long-term N addition would both suppress CH_4_ uptake; and (3) their interaction would synergistically suppress CH_4_ uptake.

## Materials and methods

### Site description

The study site was located at the Jilin Songnen Grassland Ecosystem National Observation and Research Station at the Changling Horse Breeding Farm in western Jilin province in northeastern China (44°34′25″N, 123°31′6″E; 138-176 m above sea level). The study area has a semi-arid temperate continental monsoon climate. In the past 65 years (1953-2017), average annual air temperatures ranged from 3.40°C to 7.58°C, with an average value of 5.6°C (National Meteorological Information Center). The average annual precipitation is 445 mm, with more than 80% occurring during the growing season (1953-2017, National Meteorological Information Center). The percentage distributions of precipitation pulses of different sizes and their contributions to total precipitation during the growing season over the past 65 years are shown in **Table S1**. Although precipitation pulses smaller than 5 mm were frequent (63%), they only accounted for 13.01% of the total precipitation during the growing season. In contrast, extreme pulses above 50 mm only had a frequency of 1.38%, but their contribution to the total precipitation (13.40%) was comparable to pulses below 5 mm in size.

The vegetation of the studied meadow steppe is dominated by *Leymus chinensis*. The zonal soil at the study site is classified as Salic Solonetz (World Reference Base for Soil Resources) or an Aqui-Alkalic Halosol (Chinese soil classification) (Ren et al, 2019; Shi et al., 2019). The soil is saline-alkaline with a pH value of 8.0-10.0 (Cui et al., 2021). **Table S2** shows the soil’s chemical and physical properties at 0-10 cm depth as measured in the control and long-term N addition plots before the precipitation pulse treatments were applied.

### Experimental design

In 2010, an area of 100 m × 100 m was fenced off in the *L. chinensis* meadow steppe, prohibiting grazing and mowing. In 2011, a long-term N addition experimental platform was established in the fenced area. Five blocks were set up in the experimental region, each with an area of 20 m × 10 m. Each block was then divided into two plots, each with an area of 10 m × 10 m. One plot was randomly assigned to N addition (10 g N m^-2^ yr^-1^) in each block, and the other unfertilized plot served as the control. In the long-term N addition plots, urea was applied every year in May and July at 5 g N m^-2^. In early May 2020, the intact soil columns were collected by the soil column collector (external diameter = 30.3 cm, height = 50 cm). The collector rotated and cut into 40 cm of the soil, and then the intact soil columns were collected and placed in the pots (internal diameter = 30.3 cm, height = 45 cm). Ten intact soil mesocosms (height = 40 cm) were collected from each plot (n = 100). Half of the soil mesocosms (n = 50) were used for CH_4_ flux measurement, and the other half (n = 50) were used for soil sampling. The collected soil mesocosms were placed in five blocks in the rainout shelter at the research station, with each block containing 20 mesocosms (ten control (CK) and ten N addition (NA) mesocosms). The mesocosms were buried 40 cm underground to reduce environmental interference and were watered to restore plant growth. From the middle of June to July 10^th^, the mesocosms were watered once a week with 1.44 L water (equal to a 20 mm precipitation pulse) to keep consistent soil water status. At the beginning of both May and July, urea was added at 5 g N m^-2^ to the surface of the N addition mesocosms. The precipitation pulses were conducted on July 31^st^. Based on the 65 years of historical precipitation in the study site (**Table S1**), five precipitation pulse sizes (0 mm, 5 mm, 10 mm, 20 mm, and 50 mm) were designed and applied randomly to a pair of the control (P0, P5, P10, P20, and P50) and N addition mesocosms (NP0, NP5, NP10, NP20, and NP50) in each block. The water was evenly sprayed into the P0 (0 L), P5 (0.36 L), P10 (0.72 L), P20 (1.44 L), and P50 (3.6 L) treatments using the sprayer in both control and N addition mesocosms. The CH_4_ fluxes and soil samples were collected immediately after watering (0 h), as well as at 2, 4, 8, 12, 24, 72, 144, 288, and 432 h after the precipitation pulse treatments.

### Measurement of CH_4_ fluxes

In June 2020, the open base collar (20 cm × 20 cm × 10 cm high), with a U-shaped groove (2.5 cm in width) around the upper edge, was permanently inserted into the soil of each mesocosm before the precipitation pulse experiment. The open-bottom chamber was tightly fitted to the collar during the gas sampling and sealed with water. The gas samples were collected from inside the chambers using a 100 mL plastic syringe fitted with three-way stopcocks at 0 min, 30 min, and 60 min after the chamber closure. The collected gas samples were immediately transferred to vacuumed gas sampling bags (LB-301, Dalian Delin Gas Packing Co., Ltd, Dalian, China). The concentrations of CH_4_ were analyzed within one week using the N_2_O/CH_4_ analyzer (Model 913-1054, Los Gatos Research Inc., Mountain View, CA, USA).

### Soil sampling and measurements

During each gas sampling occasion, soil temperature was measured and soil samples were collected. At the end of the precipitation pulses, aboveground vegetation in the pots was clipped to estimate aboveground biomass. Soil temperature was measured at a 5 cm layer of each soil mesocosm using a thermocouple penetration probe (Li 6400-09 TC, Li-cor Biosciences, Lincoln, NE, USA). Soil samples were taken at 0-10 cm depth of soil mesocosm using a stainless-steel corer (inner diameter of 3.3 cm). The soil samples were placed in sterile bags, transported to the laboratory with a cooler box, and stored at 4°C for subsequent analysis. The fresh soil samples were sieved using 2 mm mesh and divided into two subsamples in the laboratory. Fresh soil subsamples were analyzed for ammonium nitrogen (NH_4_^+^-N), nitrate nitrogen (NO_3_^–^-N), dissolved organic carbon (DOC), and microbial biomass carbon (MBC). The other subsamples were air-dried to determine pH and total carbon content (TC). The soil temperature, soil moisture, NH_4_^+^-N content, NO_3_^–^-N content, and pH value were measured on each sampling campaign, and the DOC, MBC, and TC were measured at the end (432 h) of precipitation pulse treatments.

The soil moisture was determined by the oven-drying method. The pH values of the soils were measured in a 1:2.5 (soil: water ratio) suspension with a PHS-3E glass pH electrode (PHS-3E, Shanghai Precision & Scientific Instrument Co., Ltd, Shanghai, China). Soil NH_4_^+^-N and NO_3_^–^-N content were extracted using 2 mol L^-1^ KCl solution by shaking for 1 h before being analyzed using a Lachat flow-injection auto-analyzer (Futura Flow Analyser, Alliance Instruments, Frepillon, France). TC was analyzed with an elemental analyzer (Vario Max CN, Elementar, Hanau, Germany). MBC was determined by the chloroform fumigation-extraction method (Vance et al., 1987). Extractable organic C in the fumigated and unfumigated samples was measured using an elemental analyzer. The MBC was calculated as the differences in DOC in the soil extracts between the fumigated and unfumigated samples. The amount of organic carbon in the un-fumigated soil extracts was used as DOC (Zhao et al., 2016). The AGB was determined by oven-drying, and harvested biomass was oven-dried at 65°C to a constant mass when weighed.

### Statistical analysis

The CH_4_ fluxes were calculated from the change in CH_4_ concentrations with time (Eq. S1). Cumulative CH_4_ fluxes were linearly and sequentially accumulated from the fluxes between every two adjacent measurement intervals (Eq. S2). The impact-treatment is the relative effects of precipitation pulses of different sizes on CH_4_ fluxes (average or cumulative) from 0 mm pulse (Eq. S3).

Two-way ANOVA was conducted to examine the effects of precipitation pulses, N addition, and their interactions on CH_4_ fluxes as well as biotic and abiotic factors. Multiple comparisons were determined using Tukey’s HSD test at a probability level of 95% (*P* < 0.05). The correlations between CH_4_ fluxes (average and cumulative) and average soil moisture were tested using a linear model. Binary linear functions were used to test the dependence of CH_4_ fluxes on soil moisture and soil temperature in each treatment. A quadratic equation was developed to describe the relationship between CH_4_ fluxes (average and cumulative) and precipitation pulse sizes. The structural equation model (SEM) was performed to analyze the direct and indirect effects of precipitation pulse and N addition on cumulative CH_4_ fluxes using the lavaan package (Rosseel, 2012). The chi-square (χ^2^) test (*P* > 0.05), comparative fit index (CFI) > 0.9, and standardized root mean-square-residual (SRMR) value < 0.08 were used to indicate if the SEM models fit well. Statistical analyses were conducted using R Statistical Software (Version 4.1.2, R Corporation, Vienna, Austria) and IBM SPSS Statistics (IBM SPSS Statistics 25.0, IBM Corporation, Chicago, IL, USA). Results were presented as mean ± 1 standard error (SE). The graphics were drawn using OriginPro 2018 software (OriginPro 2018, OriginLab Corporation, Northampton, MA, USA).

## Results

### Effects of precipitation pulses and N addition on biotic and abiotic factors

**Figure 1:**
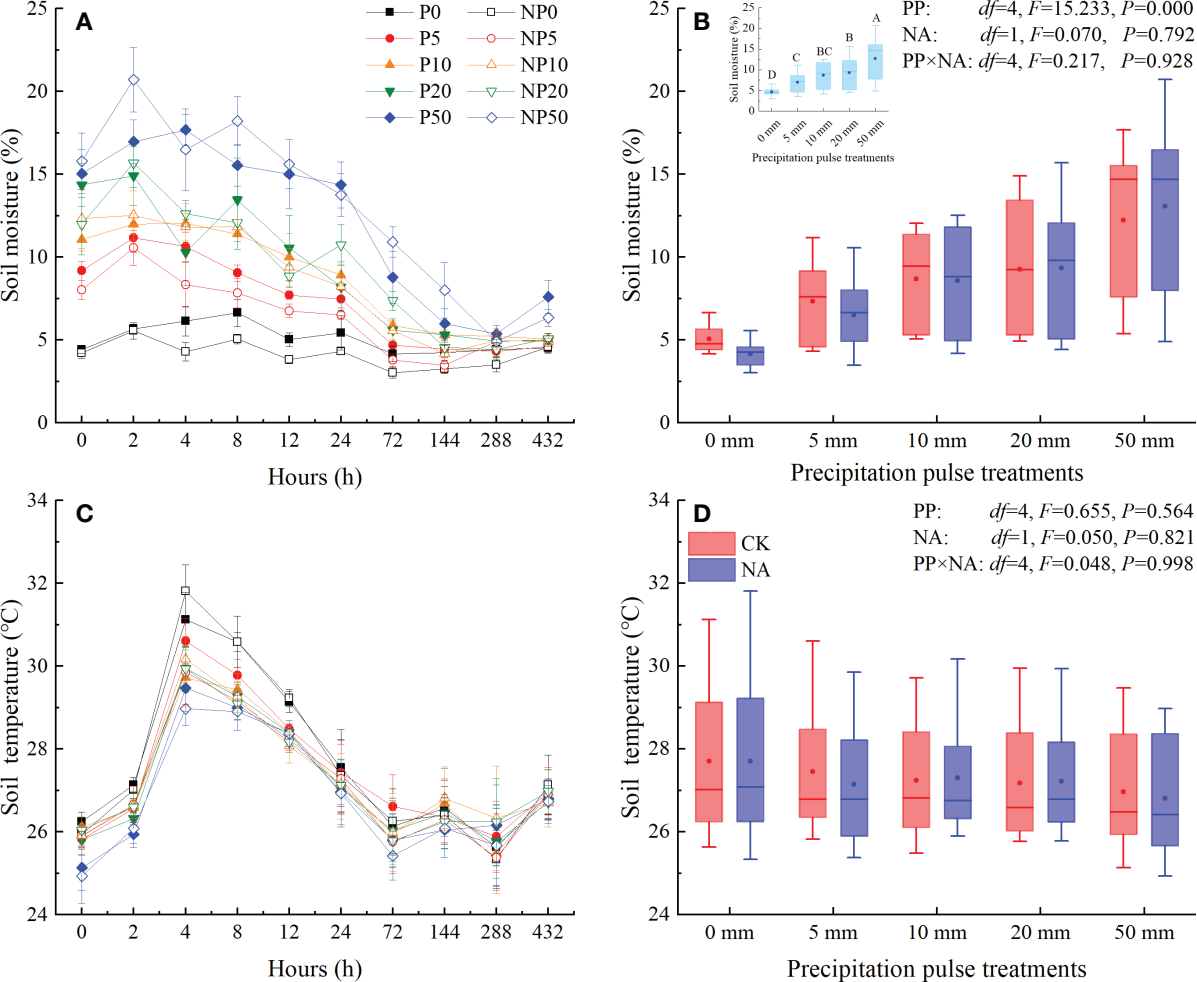
Responses of soil moisture at 0-10 cm depth (**(A)**: temporal dynamic; **(B)**: average soil moisture) and soil temperature at 5 cm depth (**(C)**: temporal dynamic; **(D)**) average soil temperature) to precipitation pulses and N addition treatments. The inserted graph in panel B (light blue column) shows the differences in average soil moisture among the precipitation pulses. Boxplots show the median (lines within the box) and interquartile range (box boundaries). Whiskers extend to the most extreme data point within 1.5 × (75-25%) data range. The solid point represents the mean value. Different capital letters (above each boxplot) denote significant differences among the precipitation pulses.

The precipitation pulse (PP) treatments caused an immediate increase in soil moisture that was related to the PP sizes (**Figure 1A**). Soil moisture peaked at around 2-4 h and then decreased until the end of precipitation pulse treatments. The PP treatments of 5 mm, 10 mm, 20 mm, and 50 mm significantly enhanced the average soil moisture (*P* = 0.000, *df* = 4; **Figure 1B**), which quadratically increased with PP size in both control and N addition treatments (all *P* = 0.000, *df* = 24; **Figure S1**). N addition (NA) as well as the interaction of PP and NA, however, did not substantially alter the average soil moisture (**Figure 1B**). Soil temperatures had significant temporal dynamics in the P0 and NP0 treatments, showing a unimodal trend (**Figure 1C**). PP, NA, and their interaction, however, had no significant effect on the temporal dynamics and average soil temperature (**Figure 1C, D**).

**Figure 2:**
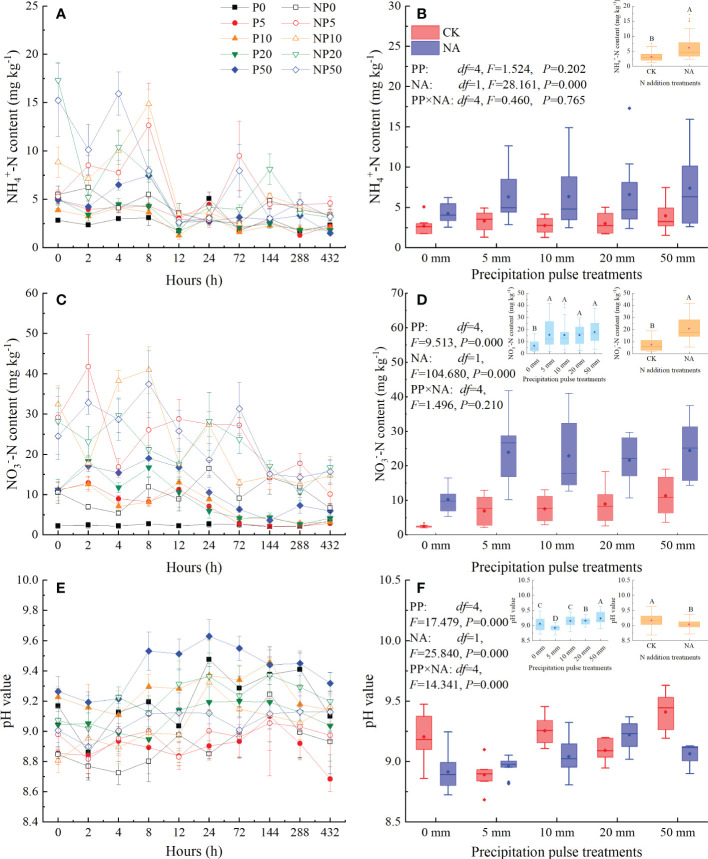
Responses of NH_4_^+^-N content (**(A)**: temporal dynamic; **(B)**: average NH_4_^+^-N content), NO_3_^−^-N content (**(C)**: temporal dynamic; **(D)**: average NO_3_^−^-N content), and pH value (**(E)**: temporal dynamic; **(F)**: average pH value) at 0-10 cm depth to precipitation pulses and N addition treatments. The inserted graphs (orange column) in panels B, D, and F show the differences in average NH_4_^+^-N content, average NO_3_^−^-N content, and average pH value between the control and N addition treatments. The inserted graphs (light blue column) in panels D and F show the differences in average NO_3_^−^-N content and average pH value among the precipitation pulses. Boxplots show the median (lines within the box) and interquartile range (box boundaries). Whiskers extend to the most extreme data point within 1.5 × (75-25%) data range. The solid point represents the mean value. Different capital letters (above each boxplot) denote significant differences among the precipitation pulses or between the control and N addition treatments.

The PP treatments significantly affected the temporal dynamics of NH_4_^+^-N content (**Figure 2A**), NO_3_^–^-N content (**Figure 2C**), and pH value (**Figure 2E**). The peaks of NH_4_^+^-N content, NO_3_^–^-N content, and pH mainly occurred at 0-8 h, 2-12 h, and 24-144 h after the PP treatments, respectively. All sizes of PP significantly increased average NO_3_^–^-N content (*P* = 0.000, *df* = 4; **Figure 2D**) but not average NH_4_^+^-N content (**Figure 2B**). The 20 mm and 50 mm PP treatments significantly increased the pH, while the 5 mm PP treatment significantly decreased the pH (*P* = 0.000, *df* = 4; **Figure 2F**). NA significantly increased NH_4_^+^-N content (*P* = 0.000, *df* = 1; **Figure 2B**) and NO_3_^–^-N content (*P* = 0.000, *df* = 1; **Figure 2D**), but significantly decreased the pH (*P* = 0.000, *df* = 1; **Figure 2F**). The interaction of PP and NA significantly affected the pH (*P* = 0.000, *df* = 4; **Figure 2F**) but had no significant effects on NH_4_^+^-N content (**Figure 2B**) or NO_3_^–^-N content (**Figure 2D**).

**Figure 3:**
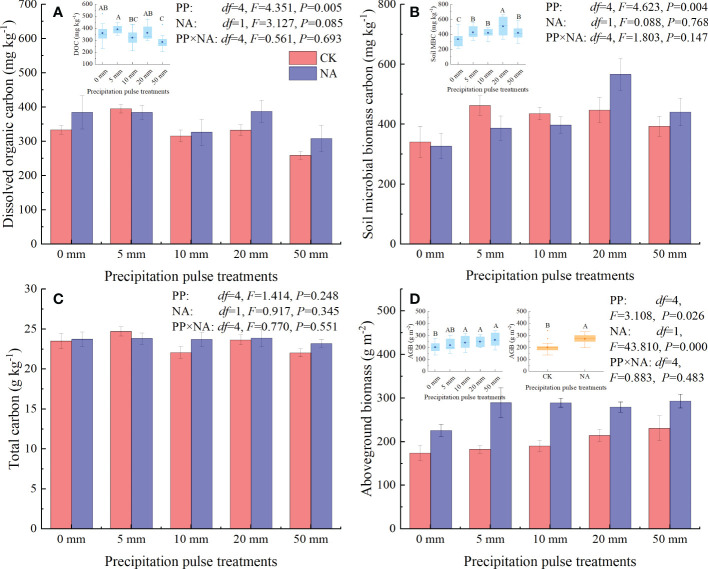
Responses of dissolved organic carbon (DOC, **A**), soil microbial biomass carbon (MBC, **B**), total carbon (TC, **C**), and aboveground biomass (AGB, **D**) to precipitation pulses and N addition treatments. The inserted graphs (light blue column) in panels **A, B, D** show the differences in DOC, MBC, and AGB among the precipitation pulses. The inserted graphs (orange column) in panel D show the differences in AGB between the control and N addition treatments. Boxplots show the median (lines within the box) and interquartile range (box boundaries). Whiskers extend to the most extreme data point within 1.5 × (75-25%) data range. The solid point represents the mean value. Different capital letters (above each boxplot) denote significant differences among the precipitation pulses treatments or between the control and N addition treatments.

At the end of precipitation treatment, PP treatments significantly affected the DOC content (*P* = 0.005, *df* = 4; **Figure 3A**), MBC content (*P* = 0.004, *df* = 4; **Figure 3B**), and AGB (*P* = 0.026, *df* = 4; **Figure 3D**), but not the TC (**Figure 3C**). Compared with the 0 mm PP treatment, 50 mm PP treatment significantly decreased DOC content (**Figure 3A**), while the 10 mm, 20 mm, and 50 mm PP treatments significantly increased MBC content and AGB (**Figure 3B, D**). NA significantly increased AGB (*P* = 0.000, *df* = 1; **Figure 3D**), but did not substantially alter DOC content (marginal effect, *P* = 0.085, *df* = 1; **Figure 3A**), MBC (**Figure 3B**), and TC (**Figure 3C**). The interaction of PP and NA had no significant effect on the DOC, MBC, TC, or AGB (**Figure 3**).

### Effects of precipitation pulses and N addition on CH_4_ fluxes

**Figure 4:**
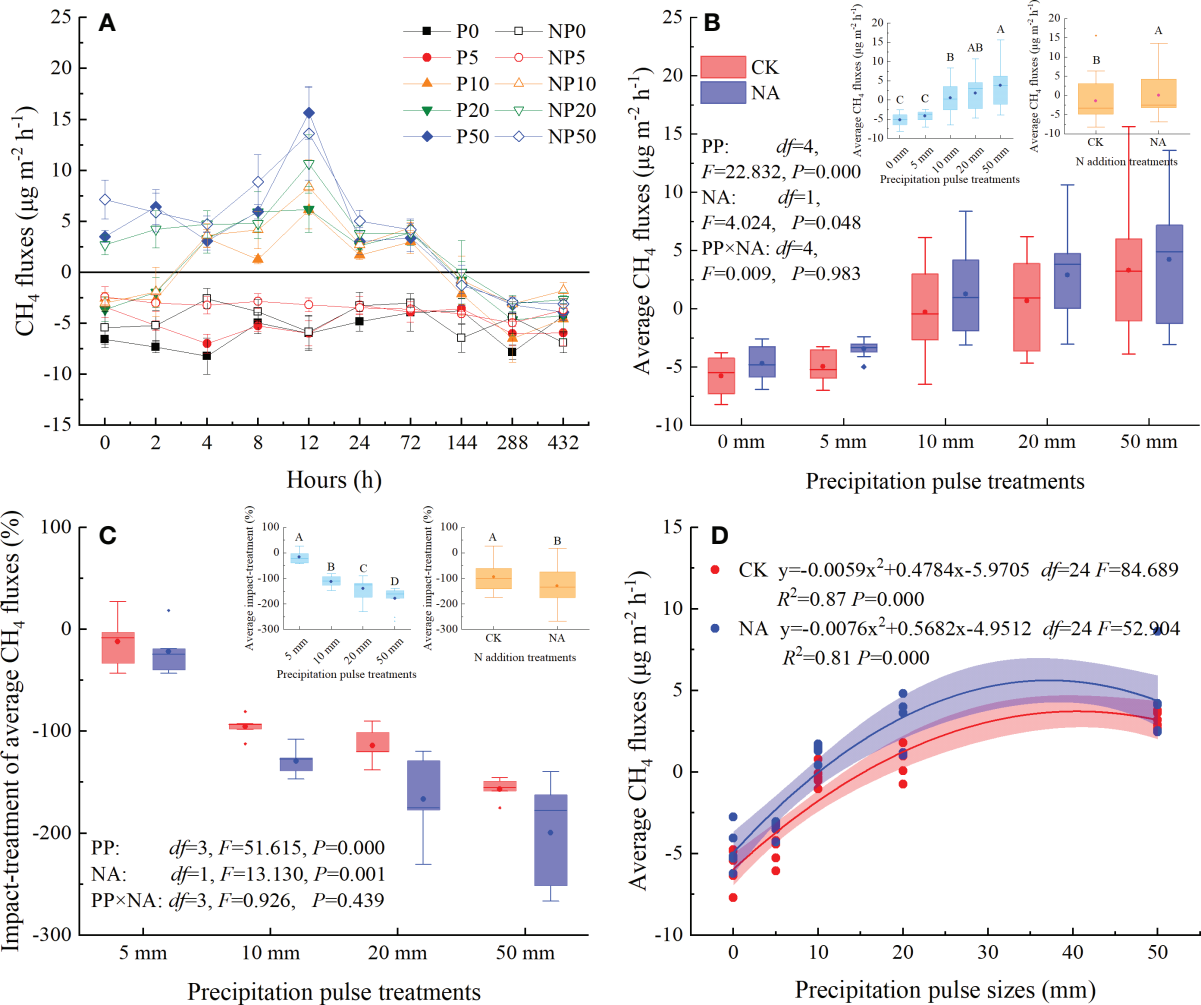
Responses of CH_4_ fluxes (**(A)**: temporal dynamic; **(B)**: average flux; **(C)**: impact-treatment of average CH_4_ flux) to precipitation pulses and N addition treatment, and the relationship between average CH_4_ fluxes and precipitation pulse sizes **(D)**. The inserted graphs (light blue column) in panels **B, C** show the differences in average CH_4_ fluxes and impact-treatment of average CH_4_ flux among the precipitation pulses. The inserted graphs (orange column) in panels **B, C** show the differences in the average CH_4_ fluxes between the control and the N addition treatments. Boxplots show the median (lines within the box) and interquartile range (box boundaries). Whiskers extend to the most extreme data point within 1.5 × (75-25%) data range. The solid point represents the mean value. Different capital letters (above each boxplot) denote significant differences among the precipitation pulses or between the control and N addition treatments.

The studied grassland acted as a sink for CH_4_ in the P0 and NP0 treatments, with fluxes ranging from -8.22 to -3.78 μg m^-2^ h^-1^ and -6.91 to -2.57 μg m^-2^ h^-1^, respectively (**Figure 4A**). The 5 mm PP treatment slightly suppressed CH_4_ uptake and average CH_4_ fluxes, but not at statistically significant levels (**Figures 4A, B**). The 10 mm, 20 mm, and 50 mm PP treatments, however, substantially changed the temporal dynamics and the source-sink relationship of CH_4_ fluxes (**Figure 4A**). The 10 mm PP treatment shifted CH_4_ fluxes from sinks to sources within 2 to 4 h, while 20 mm and 50 mm PP treatments immediately triggered the release of CH_4_ (**Figure 4A**). The CH_4_ fluxes primarily peaked at 12 h and relatively decreased the CH_4_ uptake by 203-362% and 243-333% compared with P0 and NP0 treatments, respectively (**Figure 4A**). After that, CH_4_ releases decreased and absorption resumed at 144 h until the end of PP treatments (**Figure 4A**).

The PP significantly affected the average CH_4_ fluxes (*P* = 0.000, *df* = 4; **Figure 4B**). More specifically, the 10 mm, 20 mm, and 50 mm PP treatments significantly enhanced the average CH_4_ fluxes (109-171%), but the 5 mm PP treatment had no significant effect (**Figure 4B**). The relative effect of PP treatment on CH_4_ fluxes increased significantly with increasing PP sizes (*P* = 0.000, *df* = 3; **Figure 4C**). The relationship between the average CH_4_ fluxes and PP sizes could be fitted by a quadratic equation, explaining the 87% (*P* = 0.000, *df* = 24) and 81% (*P* = 0.000, *df* = 24) variation in the average CH_4_ fluxes in the control and N addition treatments, respectively (**Figure 4D**). N addition significantly enhanced the average CH_4_ fluxes (*P* = 0.048, *df* = 1; **Figure 4B**) and the impact-treatment of average CH_4_ fluxes (*P* = 0.001, *df* = 1; **Figure 4C**). The interaction of PP and NA, however, had no significant influence on average CH_4_ fluxes (**Figure 4B**) or the impact-treatment of average CH_4_ fluxes (**Figure 4C**).

**Figure 5:**
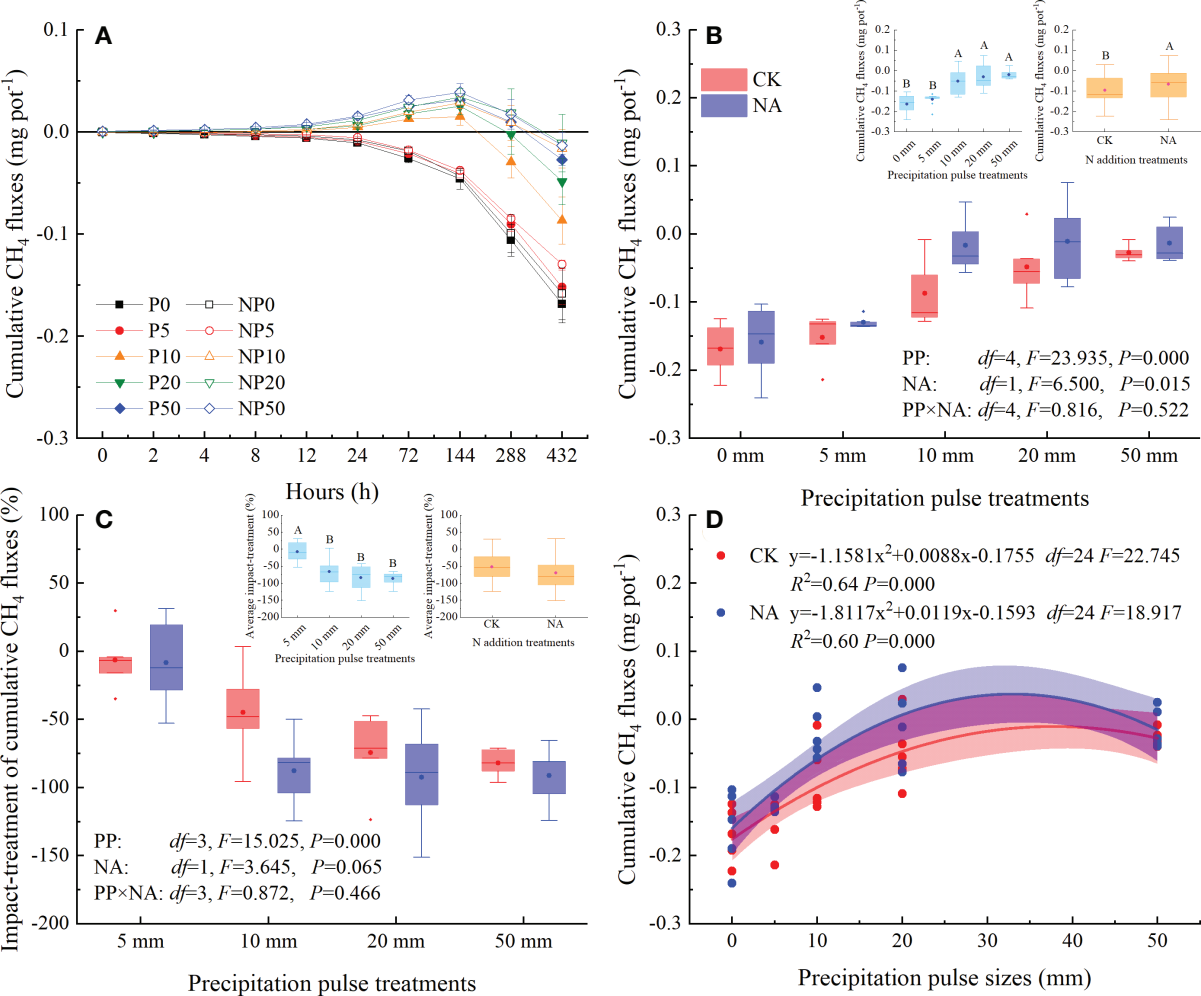
Responses of the cumulative CH_4_ fluxes (**(A)**: temporal dynamic; **(B)**: average cumulative fluxes; **(C)**: impact-treatment of cumulative CH_4_ flux) to precipitation pulses and N addition, and the relationship between cumulative CH_4_ fluxes and precipitation pulse sizes **(D)**. The inserted graphs (light blue column) in panels **B, C** show the differences in cumulative CH_4_ fluxes and impact-treatment of cumulative CH_4_ flux among the precipitation pulses. The inserted graph (orange column) in panel **B** shows the differences in cumulative CH_4_ fluxes between the control and N addition treatments. Boxplots show the median (lines within the box) and interquartile range (box boundaries). Whiskers extend to the most extreme data point within 1.5 × (75-25%) data range. The solid point represents the mean value. Different capital letters (above each boxplot) denote significant differences among the precipitation pulses or between the control and N addition treatments.

Cumulative CH_4_ fluxes showed a decreasing trend following the 0 mm and 5 mm PP treatments, as CH_4_ continued to be absorbed from the atmosphere by the soil (**Figures 4A**, **5A**). The 10 mm, 20 mm, and 50 mm PP treatments significantly increased the cumulative CH_4_ fluxes, reaching the highest cumulative fluxes at 144 h (**Figure 5A**). After that, cumulative CH_4_ fluxes were reduced and the areas were converted into CH_4_ sinks. During the experimental period, cumulative CH_4_ fluxes ranged from -0.17 to -0.03 mg CH_4_ pot^-1^ and -0.16 to -0.02 mg CH_4_ pot^-1^ in the control and N addition treatments, respectively (**Figure 5A**).

The PP treatments significantly affected the cumulative CH_4_ fluxes (*P* = 0.000, *df* = 4; **Figure 5B**) and the impact-treatment of cumulative CH_4_ fluxes (*P* = 0.000, *df* = 3; **Figure 5C**). The 10 mm, 20 mm, and 50 mm PP treatments significantly reduced the absorption of CH_4_ (68-88%), with a higher relative effect on cumulative CH_4_ fluxes; in contrast, the 5 mm PP treatment did not have any effect (**Figure 5B, 5C**). There was a significant quadratic relationship between the cumulative CH_4_ fluxes and PP sizes, which could explain the 64% (*P* = 0.000, *df* = 24) and 60% (*P* = 0.000, *df* = 24) variation in the cumulative CH_4_ fluxes in the control and N addition treatments, respectively (**Figure 5D**). NA significantly increased the cumulative CH_4_ fluxes (*P* = 0.015, *df* = 1; **Figure 5B**), but had a marginal effect on the impact-treatment of cumulative CH_4_ fluxes (*P* = 0.065, *df* = 1; **Figure 5C**). The interaction of PP and NA, however, did not significantly affect the cumulative CH_4_ fluxes (**Figure 5B**) and the impact-treatment of cumulative CH_4_ fluxes (**Figure 5C**).

### Dependences of temporal dynamics of CH_4_ fluxes on soil moisture and soil temperature

The binary linear model showed that the temporal dynamics of CH_4_ fluxes were mainly driven by soil moisture and soil temperature after the PP treatments (**Table 1**). Only the P0 and NP0 treatments had no significant relationship between CH_4_ fluxes and soil moisture and soil temperature (**Table 1**). The CH_4_ fluxes were significantly positively correlated with soil moisture and soil temperature in the NP5 treatment (*P* < 0.01, *df* = 49), but were significantly negatively correlated with soil moisture in the P5 treatment (*P* < 0.05, *df* = 42). The CH_4_ fluxes from control and N addition treatments were both significantly positively correlated with soil moisture and soil temperature in the 10 mm (both *P* < 0.01), 20 mm (both *P* < 0.01), and 50 mm (both *P* < 0.001) PP treatments. The degree of fitting increased with increasing PP sizes, explaining the 10-34% and 18-28% change in the temporal dynamics of CH_4_ fluxes in the control and N addition treatments, respectively (**Table 1**).

**Table 1:** Dependency of CH_4_ fluxes (*F*) on soil moisture (*SM*, 0-10 cm depth) and soil temperature (*ST*, 5 cm depth) after precipitation pulses and long-term N addition treatments.

Treatments	Function	*df*_num_	*df*_den_	*F*	*R* ^2^	*P*
Control						
P0						NS
P5	*F* = 0.950*SM* - 0.141*ST* - 6.030	2	40	1.636	0.10	<0.05
P10	*F* = 0.607*SM* + 0.480*ST* - 18.240	2	45	5.337	0.18	<0.01
P20	*F* = 0.006*SM* + 1.903*ST* - 50.724	2	43	6.879	0.18	<0.01
P50	*F* = 0.608*SM* + 0.895*ST* – 29.370	2	44	9.381	0.34	<0.001
N addition						
P0						NS
P5	*F* = 0.196*SM* + 0.927*ST* - 28.882	2	47	1.877	0.18	<0.01
P10	*F* = 0.301*SM* + 1.222*ST* - 37.480	2	47	2.617	0.22	<0.01
P20	*F* = 0.553*SM* + 0.871*ST* - 28.269	2	44	8.415	0.25	<0.001
P50	*F* = 0.489*SM* + 0.673*ST* - 28.269	2	43	9.083	0.28	<0.001

df_num_: df of numerator; df_den_: df of denominator.

NS, not significant.

### Relationships between cumulative CH_4_ fluxes with biotic and abiotic factors

**Figure 6:**
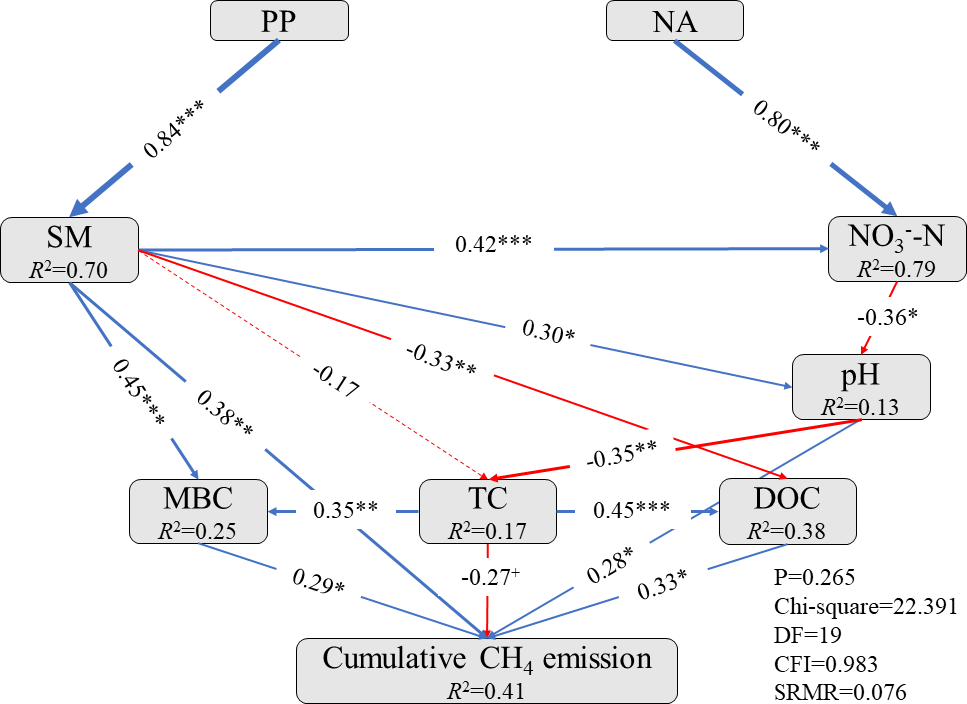
Structural equation model (SEM) performed to examine the direct and indirect effects of precipitation pulses and long-term N addition on cumulative CH_4_ fluxes. The blue arrows indicated positive effects, while the red arrows indicated negative effects. The solid arrows indicated significant paths (*P* < 0.05). Conversely, the dotted lines indicated insignificant paths (*P* > 0.05). Arrow width represented the strength of the relationship. Values associated with solid arrows represent standardized path coefficients. *R* ^2^ values represent the proportion of the variance explained for each endogenous variable. Significance levels are as follows: ^+^, *P* < 0.1; *, *P* < 0.05; **, *P* < 0.01; ***, *P* < 0.001. Goodness-of-fit statistics are shown below the model. PP, precipitation pulse; NA, N addition; SM, soil moisture; NO_3_^–^-N, NO_3_^–^-N content; pH, pH value; MBC, microbial biomass carbon; TC, total carbon; DOC, dissolved organic carbon.

Structural equation model (SEM) analysis indicated that N addition significantly increased the NO_3_^–^-N content (*P* < 0.001), thereby decreasing the soil pH (*P* < 0.01; **Figure 6**). The pH change directly increased the cumulative CH_4_ fluxes (*P* < 0.05) and indirectly enhanced the MBC (*P* < 0.01) and DOC (*P* < 0.001) through decreased TC (*P* < 0.01). The MBC and DOC significantly increased the cumulative CH_4_ fluxes (both *P* < 0.05), while the TC had a marginal effect on cumulative CH_4_ fluxes (*P* < 0.1). The precipitation pulses significantly increased the soil moisture (*P* < 0.001), which directly increased the cumulative CH_4_ fluxes (*P* < 0.01). Additionally, soil moisture positively affected NO_3_^–^-N content (*P* < 0.001), pH (*P* < 0.05), and MBC (*P* < 0.001), but a negative effect on DOC (*P* < 0.01) and TC (*P* > 0.05); this, in turn altered the cumulative CH_4_ fluxes. All factors jointly explained the 41% variation in cumulative CH_4_ fluxes that were observed (**Figure 6**).

## Discussion

### The suppression effect of precipitation pulses on cumulative CH_4_ fluxes

The effects of precipitation pulses on CH_4_ fluxes are very complex. Previous studies have shown that precipitation pulses could stimulate (Harms and Grimm, 2012; Petrakis et al., 2017), suppress (Zhao et al., 2017; Ren et al., 2019), or not significantly affect CH_4_ fluxes (Mariko et al., 2007; Ni et al., 2019). The effects mainly depended on the precipitation pulse size, ecosystem type, and soil moisture status (Harms and Grimm, 2012; Kim et al., 2012; Petrakis et al., 2017; Zhao et al., 2017; Ni et al., 2019). Without manipulated precipitation, the studied grassland acted as a net sink of CH_4_ (**Figures 4A**, **5A**), which is consistent with the findings of previous studies (Dalal and Allen, 2008; Wang et al., 2014b; Yu et al., 2017; Zhao et al., 2017). The average rate of CH_4_ absorption of the studied grassland (-5.23 ± 0.36 μg m^-2^ h^-1^, **Figure 4B**) was, however, much lower than that of the semi-arid grassland from northeast China (-74.31 ± 62.51 μg m^-2^ h^-1^) as well as the Qinghai-Tibetan Plateau (-31.29 ± 21.78 μg m^-2^ h^-1^) (Wang et al., 2014b). As expected, the large (10 mm, 20 mm, and 50 mm) precipitation pulses had a negative pulsing effect on CH_4_ fluxes and significantly suppressed CH_4_ absorption, but the small (5 mm) precipitation pulse did not significantly alter it (**Figure 5B**).

Without water supplementation (0 mm PP treatment), the soil was arid and soil moisture was relatively stable (**Figure 1A**). Low soil moisture limited the activity of methanotrophs due to water stress, and the absorption rate of CH_4_ was, therefore, very low (Le Mer and Roger, 2001; Borken et al., 2006; Aronson et al., 2019). Although the 5 mm PP treatment substantially increased average soil moisture (**Figure 1B**), it did not significantly alter the temporal dynamics or cumulative CH_4_ fluxes (**Figures 4A**, **5B**). Ni et al. (2019) also confirmed that short-term precipitation pulses did not alter CH_4_ fluxes in a forest ecosystem, suggesting that small precipitation changes do not alter O_2_ in soil pore spaces enough to affect CH_4_ fluxes. As expected, the large precipitation pulses (10 mm, 20 mm, and 50 mm) altered the source-sink relationship of CH_4_, converting CH_4_ fluxes of the studied grassland from a sink to a source (**Figures 4A**, **5A**), which were consistent with an *in situ* field study (Ren et al., 2019). The temporal dynamics of CH_4_ fluxes were controlled by the soil moisture and soil temperature following the precipitation pulses (**Table 1**). While the precipitation pulses significantly increased soil moisture, they did not alter soil temperature (**Figure 1**). The changes in soil moisture dynamics caused by precipitation pulses, therefore, may be responsible for the changes in CH_4_ fluxes. In this study, there were several potential mechanisms by which precipitation pulses could have triggered the CH_4_ source-sink conversion. Firstly, the infiltration of soil water caused by the precipitation pulses could displace CH_4_ trapped in the soil pore space and release it to the atmosphere, especially in the case of the 50 mm PP treatment. Secondly, a large precipitation pulse could increase the soil water availability and alleviate water limitation, decreasing the redox potential and availability of O_2_ in favor of anaerobic processes, thereby promoting methanogenic activity and suppressing CH_4_ oxidation (Harms and Grimm, 2012; Kim et al., 2012). Thirdly, the increased availability of water could stimulate microbial, specifically methanogen, biomass (Huang et al., 2015; Venturini et al., 2022). Fourthly, the availability of substrates before PP treatments was accumulated through microbial metabolism, soil aggregates shattering, and organisms death, which would be rapidly utilized by methanogen under these increased soil moisture conditions (Unger et al., 2010; Harms and Grimm, 2012; Kim et al., 2012; Lado-Monserrat et al., 2014). Though precipitation pulses break the balance between the CH_4_ production and consumption and change the source-sink relationship of CH_4_ fluxes through physical and biological processes (**Figure 4A**), they cannot permanently change the nature of grassland as a CH_4_ sink (**Figure 5B**). In the context of global change, however, it appears that the sink strength of grassland ecosystems will decrease with the increase of heavy precipitation pulses in the future (Zhao et al., 2017; Ren et al., 2019).

**Figure 7:**
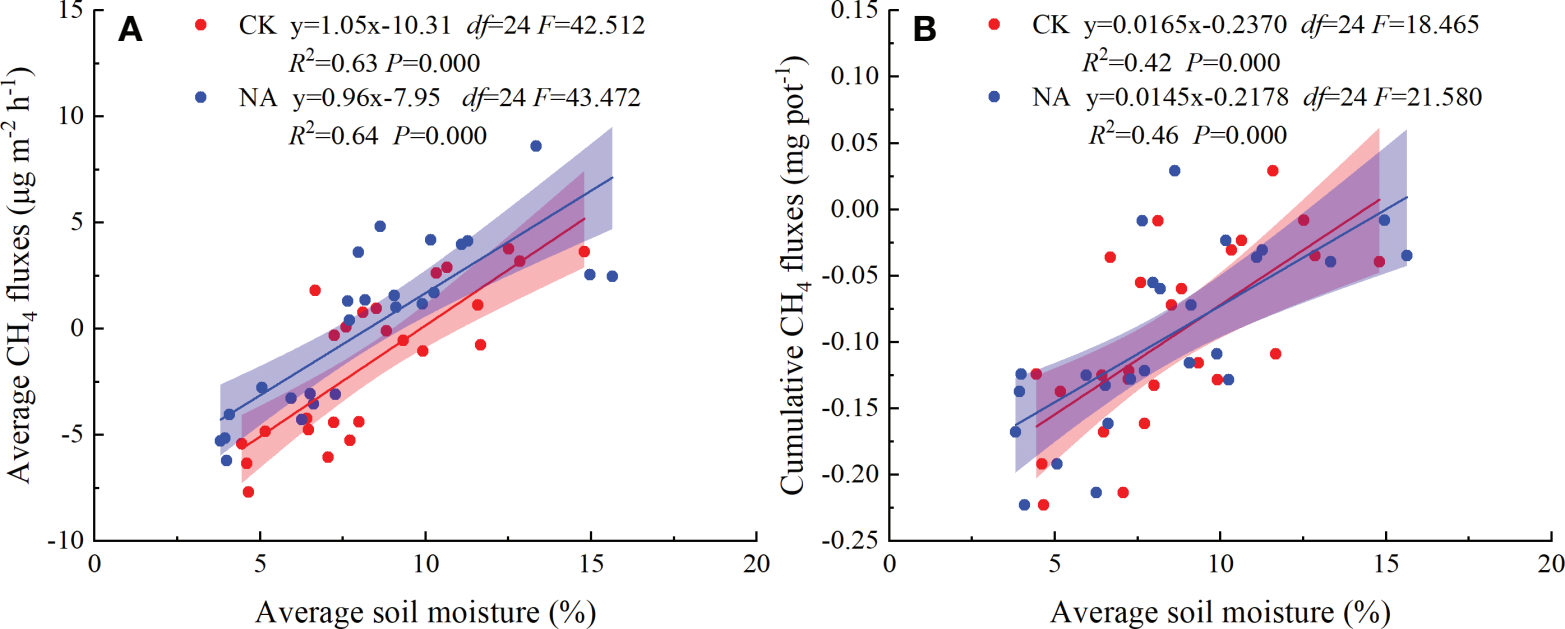
The relationship between CH_4_ fluxes (**(A)**: average fluxes; **(B)**: cumulative fluxes) and average soil moisture after the precipitation pulses and long-term N addition treatments.

For the first time it was demonstrated that cumulative CH_4_ fluxes increased quadratically with precipitation pulse size in both the control and N addition treatments (**Figure 5D**). These results further suggest that precipitation pulses suppress CH_4_ uptake by controlling soil moisture (**Figures 5B**, **7B**, **S1**). Average soil moisture in the 0-10 cm layer quadratically increased with precipitation pulse size after precipitation treatments (all *P* = 0.000, *df* = 24; **Figure S1**). Contrary to expectations, the optimal relationship between soil moisture and precipitation pulse size was not linear. Extreme precipitation pulses (20 mm and 50 mm) caused soil moisture to penetrate deeper and significantly increased average soil moisture in the 10-30 cm soil layer (*P* = 0.000, *df* = 4; **Figure S2**). The 10 mm, 20 mm, and 50 mm precipitation pulses significantly increased aboveground biomass (**Figure 3D**), which then also consumed a large amount of soil water. The increased aboveground biomass can also increase soil water by reducing soil evaporation, but the effect is largely unknown. Soil moisture infiltration and plant growth together decreased soil moisture in the 0-10 cm soil layer when larger precipitation pulse treatments were applied, resulting in a quadratic increase in average soil moisture with precipitation pulse size. Cumulative CH_4_ fluxes increased linearly with average soil moisture due to the different responses of gas diffusion and activity of microbe to the increased soil moisture (**Figure 7**). The small precipitation pulses did not alter soil water contents and O_2_ concentrations enough to affect the CH_4_ oxidation environment (Ni et al., 2019). The large precipitation pulses significantly inhibited the soil gases diffusion participating in CH_4_ oxidation and suppressed CH_4_ oxidation (Liu et al., 2008; Zhao et al., 2017). At the same time, the large precipitation pulses decreased the redox potential, created a saturated soil condition and lasted for a few days, which were conducive to methanogenesis (Harms and Grimm, 2012; Decock and Six, 2013; Petrakis et al., 2017). This study saw the release of CH_4_ from the soil to the atmosphere under these conditions (**Figure 4A**), confirming that large precipitation pulses (10 mm, 20 mm, and 50 mm) stimulated the activity of methanogens (Ren et al., 2019). The decreased soil gases diffusion participating in CH_4_ oxidation and increased activity of methanogens, therefore, led to a decrease in CH_4_ uptake with increasing soil moisture. Ecologists have confirmed that CH_4_ uptake decrease with increasing soil moisture in grassland ecosystems (Jiang et al., 2010; Shrestha et al., 2012; Liu et al., 2021a; Wu et al., 2021). In conclusion, precipitation pulses suppressed CH_4_ uptake by increasing soil moisture and exhibited a quadratic relationship with the cumulative CH_4_ fluxes.

### The suppression effect of long-term N addition on cumulative CH_4_ fluxes

Effects of N addition on CH_4_ fluxes have been extensively studied in many ecosystems (Aronson and Helliker, 2010; Deng et al., 2020; Wu et al., 2022). As expected, N addition significantly suppressed the absorption of CH_4_ in the studied grassland ecosystem (**Figures 4B**, **5B**), consistent with the results of the meta-analyses (Aronson and Helliker, 2010). In the present study, N addition significantly increased the NH_4_^+^-N content (**Figure 2B**) and NO_3_^–^N content (**Figure 2D**), along with aboveground biomass (**Figure 3D**), while significantly decreasing the pH value (**Figure 2E**). N addition, therefore, could decrease the absorption of CH_4_ or increase the production of CH_4_ through changing amounts of NH_4_^+^-N, NO_3_^–^-N, and AGB, as well as altering pH value, suppressing CH_4_ uptake (Fang et al., 2014; Yang et al., 2017; Chen et al., 2019; Kong et al., 2019; Ren et al., 2019).

There are multiple underlying mechanisms for N addition suppressing CH_4_ uptake. First, the increased NH_4_^+^-N competes with CH_4_ for methane monooxygenase (MMO), which decreased the combination point of MMO to CH_4_, thereby reducing the oxidation of CH_4_ (Schnell and King, 1994). Second, NH_4_^+^-N is oxidized to hydroxylamine (NH_2_OH) and nitrite (NO2--N) by CH_4_ monooxygenase or ammonia-oxidizing microorganisms, which has a toxic effect on methanotrophs (Bodelier, 2011). Third, the NH_4_^+^-N and NO_3_^–^-N content in the N addition treatment were 1.96 and 2.77 times greater than that of the unfertilized treatment, respectively (**Figure 2D**), resulting in osmotic stress and suppressing the activity of methanotrophs (Bodelier and Laanbroek, 2004; Saari et al., 2004; Yang et al., 2017). Four, the increased AGB could allocate more C to promote root exudates, which would improve substrate availability for methanogens (Waldo et al., 2019). Additionally, N addition could enhance litter mass input to soil and nutrient return from litter decomposition by increasing AGB, thereby alleviating the C limitation on methanogens (Gong et al., 2015; Ren et al., 2019). As a result, methanogens enhanced the CH_4_ production, which in turn offset the absorption of CH_4_ and suppressed CH_4_ uptake (Ren et al., 2019). It was found that cumulative CH_4_ fluxes were marginally significant positively correlated with the changes in average NH_4_^+^-N (*P* = 0.114, *df* = 49; **Figure S3A**) and NO_3_^–^-N content (*P=* 0.077, *df* = 49; **Figure S3B**), as well as AGB (*P=* 0.074, *df* = 49; **Figure S3C**). This implies that N addition could decrease the absorption of CH_4_ by increasing NH_4_^+^-N content and NO_3_^–^-N content, and/or increase the production of CH_4_ by increasing AGB, ultimately suppressing CH_4_ uptake.

N addition significantly reduced soil pH value by 0.13 units (**Figure 2F**), half of the average global level for terrestrial ecosystems (Tian and Niu, 2015). A significant positive correlation between cumulative CH_4_ fluxes and pH value was detected (*P=* 0.042, *df*=49; **Figure S3D**), consistent with the findings of Ren et al. (2019). Contrary to expectations, the decreased pH enhanced CH_4_ uptake in saline-alkaline soils, possibly due to reduced pH alleviating the physiological stress of saline-alkaline conditions on methanotrophs. In summary, N addition suppressed CH_4_ uptake not by reducing pH value, but by increasing NH_4_^+^-N content, NO_3_^–^-N content, and AGB.

### No interactive effect of precipitation pulses and N addition on cumulative CH_4_ fluxes

Both the precipitation pulses and N addition significantly suppressed CH_4_ uptake (**Figures 4B**, **5B**). In contrast, the precipitation pulses and N addition together had no interactive effect on CH_4_ uptake (**Figures 4B**, **5B**). Several potential mechanisms could explain this result. First, cumulative CH_4_ emissions after precipitation pulses were significantly affected by soil moisture (*P* < 0.001, *df* = 49), pH (*P* < 0.001, *df* = 49), and DOC (*P* < 0.05, *df* = 49; **Figure S4**). There were no significant interactive effects between precipitation pulses and N addition on soil moisture (**Figure 1B**) and DOC (**Figure 3A**). The precipitation pulses and N addition had an interactive effect on the pH (**Figure 2F**). The pH value, however, had opposite responses to precipitation pulses and N addition (**Figure 2F**), as precipitation pulses significantly increased the pH, whereas N addition significantly decreased it (**Figure 2F**). Precipitation pulses and N addition, therefore, could not interactively affect CH_4_ uptake by interactively affecting soil moisture, pH, and DOC. Second, precipitation pulses suppressed CH_4_ uptake by increasing soil moisture, whereas N addition suppressed CH_4_ uptake by increasing NH_4_^+^-N, NO_3_^–^-N, and AGB. Precipitation pulses and N addition inhibited the absorption of CH_4_ through different pathways. Third, the structural equation model showed that pH value was a key factor in precipitation pulses, and N addition interactively affected CH_4_ fluxes. Precipitation pulses significantly increased pH by increasing soil moisture, while N addition decreased pH through increasing NO_3_^–^-N content (**Figure 6**). The precipitation pulses and N addition together, therefore, had opposite effects on pH. Additionally, N addition decreased cumulative CH_4_ fluxes by decreasing pH (total correlation coefficient: 0.100), whereas the precipitation pulses increased cumulative CH_4_ fluxes by increasing soil moisture (**Figure 6**). Precipitation pulses and N addition together, therefore, had an offset effect on cumulative CH_4_ fluxes, rather than a synergistic suppressing effect. In summary, there were no interactive effects of precipitation pulses and N addition on cumulative CH_4_ fluxes.

## Conclusions

This study evaluated the effects of precipitation pulses, N addition, and their interactions on CH_4_ fluxes as well as examined their driving mechanisms in a semi-arid meadow steppe in Northeast China. Both precipitation pulses and N addition significantly suppressed CH_4_ uptake. Precipitation pulses significantly altered the temporal dynamics of soil moisture, resulting in a negative pulse effect on CH_4_ fluxes and shifting the grassland ecosystem from a CH_4_ sink to a source. The cumulative CH_4_ fluxes increased quadratically with precipitation pulse sizes in both control and N addition treatments. N addition possibly decreases the absorption of CH_4_ by increasing NH_4_^+^-N content and NO_3_^–^-N content, or increases the production of CH_4_ by increasing aboveground biomass, ultimately inhibiting CH_4_ uptake. The plants could influence the response of CH_4_ fluxes to precipitation pulses and N addition by regulating water and substrate availability. Surprisingly, precipitation pulses and N addition had no interactive effects on CH_4_ fluxes because precipitation pulses and N addition had an offset effect on the key factor (pH) and affected CH_4_ fluxes through different pathways. The interactive effects between precipitation and N addition on CH_4_ fluxes should be further investigated in the future.

## Data availability statement

The original contributions presented in the study are included in the article/**Supplementary Material**. Further inquiries can be directed to the corresponding authors.

## Author contributions

WG: Conceptualization, Investigation, Formal analysis, Visualization, Writing – original draft, Writing – review and editing. XY: Conceptualization, Investigation, Formal analysis, Visualization, Writing – original draft, Writing – review and editing. YZ: Formal analysis, Writing – review and editing. TZ: Conceptualization, Investigation, Formal analysis, Visualization, Writing – original draft. BS: Formal analysis, Writing – original draft, Writing – review and editing. TY: Formal analysis, Visualization, Writing – review and editing. JM: Conceptualization, Formal analysis, Writing – review and editing. WX: Formal analysis, Visualization, Writing – original draft. YW: Formal analysis, Visualization, Writing – review and editing. WS: Conceptualization, Funding acquisition, Supervision, Writing – original draft, Writing – review and editing. All authors contributed to the article and approved the submitted version.
