# Supplementary material for: Suppression of methane uptake by precipitation pulses and long-term nitrogen addition in a semi-arid meadow steppe in northeast China
Source: Front Plant Sci. 2023 Jan 16;13:1071511. doi: 10.3389/fpls.2022.1071511 (PMC9884686; doi:10.3389/fpls.2022.1071511)
Supplement: Supplementary file 1 [file DataSheet_1.pdf]

Supplementary material

Suppression of methane uptake by precipitation pulses and long-term nitrogen addition in a semi-arid meadow steppe in northeast China

Weifeng Gao[1, †], Xu Yang[1, †], Yicong Zhang[1], Tianhang Zhao[1], Baoku Shi[1], Tianxue Yang[1, 2], Jianying Ma[3, *], Wanling Xu[1, 4], Yining Wu[5], Wei Sun[1, 2, *]

[1] *Institute of Grassland Science, Key Laboratory of Vegetation Ecology of the Ministry of Education, Jilin Songnen Grassland Ecosystem National Observation and Research Station, Northeast Normal University, Changchun, Jilin 130024, China*

[2] *State environmental protection key laboratory of wetland ecology and vegetation restoration, Northeast Normal University, Changchun, Jilin 130024, China*

[3] *Key Laboratory of Geographical Processes and Ecological Security in Changbai Mountains, Ministry of Education, School of Geographical Sciences, Northeast Normal University, Changchun, Jilin 130024, China*

[4] *College of Geography and Ocean Sciences, Yanbian University, Hunchun 133300, China*

[5] *College of Wildlife and Protected Area, Northeast Forestry University, Harbin 150040, China*

*** Correspondence:**

Corresponding Author: Wei Sun, Jianying Ma

E-mail address: sunwei@nenu.edu.cn (Wei Sun), majy652@nenu.edu.cn (Jianying Ma)

[†] These authors contributed equally to this work.

The CH_4 fluxes were calculated from the change in CH_4 concentrations with time, as follows:

$$F = \frac{\mathrm{d}c}{\mathrm{d}t}\frac{M}{V_0}\frac{P}{P_0}\frac{T_0}{T}H \tag{S1}$$

where F is the CH_4 flux (μg m^{-2} h^{-1}), the negative flux indicates uptake of CH_4 by the soil from the atmosphere, whereas the positive flux indicates a release of CH_4 from soil to the atmosphere; d*c*/d*t* is the change in CH_4 concentration within the chamber over time (μmol mol^{-1} h^{-1}); M is the mole mass of the CH_4 gas (g mol^{-1}); P is the atmospheric pressure (Pa); T is the average temperature (K) inside the chamber; V_0, T_0, and P_0 are the gas mole volume (m^3 mol^{-1}), absolute air temperature (K), and atmospheric pressure (Pa) under standard conditions, respectively; and H is the effective height from the soil surface to the top of the chamber (m).

Cumulative CH_4 fluxes were linearly and sequentially accumulated from the fluxes between every two adjacent intervals of the measurements, as follows:

$$C = \frac{\sum_{i=1}^{n}(F_{i+1} + F_i)}{2} \times (t_{i+1} - t_i) \times A_{pot} \quad \text{(S2)}$$

where C is the cumulative CH_4 fluxes (mg pot^{-1}); F represents CH_4 fluxes (μg m^{-2} h^{-1}); i represents the ith measurements; n represents the total times of measurements; the term (t_{i+1} - t_i) represents the hours between two adjacent times of measurements; A_{pot} represents the area of the pot.

The effects of precipitation pulses on average CH_4 fluxes and cumulative CH_4 fluxes were identified as impact-treatment:

$$\text{Impact} - \text{treatment} = \frac{(\text{Flux}_{Pi} - \text{Flux}_{P0})}{\text{Flux}_{P0}} \times 100\% \qquad \text{(S3)}$$

Impact-treatment (%) is the relative variation of average CH_4 fluxes or cumulative CH_4 fluxes; Flux_{Pi} is the average CH_4 fluxes or cumulative CH_4 fluxes at the 5 mm, 10 mm, 20 mm, and 50 mm precipitation pulses; Flux_{P0} is the average CH_4 fluxes or cumulative CH_4 fluxes at the 0 mm precipitation pulse.

Supplementary Table S1 Percentage distributions of precipitation pulses in different sizes and their contributions to total precipitation during the growing season (May-September) at the study site over the past 65 years (1953-2017).

Precipitation pulse sizes (mm)	Percentage of precipitation pulse sizes (%)	Contribution to the growing season precipitation (%)
P≤5	63.06	13.01
5≤P≤10	14.87	15.09
10≤P≤20	12.93	25.84
20≤P≤50	7.76	32.66
P≥50	1.38	13.40

Supplementary Table S2 The soil chemical and physical properties at 0-10 cm depth between control and long-term N addition plots before precipitation pulses at the study sites.

Environmental factors	Control plots	Long-term N addition plots
Soil moisture (%)	5.19 ± 0.62A	4.65 ± 0.47A
Soil temperature (℃)	26.42 ± 0.14A	26.38 ± 0.09A
pH value	9.29 ± 0.22A	9.13 ± 0.10A
NH_4^+-N content (mg kg^{-1})	3.37 ± 0.15B	6.67 ± 0.93A
NO_3^--N content (mg kg^{-1})	1.08 ± 0.12B	3.60 ± 0.33A
Dissolved organic carbon (mg kg^{-1})	336.07 ± 20.61A	377.33 ± 40.76A
Microbial biomass carbon (mg kg^{-1})	367.90 ± 34.93A	441.53 ± 45.36A
Microbial biomass nitrogen (mg kg^{-1})	10.37 ± 2.47A	18.17 ± 3.33A
Total carbon (g kg^{-1})	24.62 ± 1.05A	23.54 ± 0.56A
Total nitrogen (g kg^{-1})	1.58 ± 0.21A	1.64 ± 0.11A
C/N ratio	16.37 ± 1.56A	14.65 ± 1.11A

Different capital letters denote significant differences between control and N addition treatments.

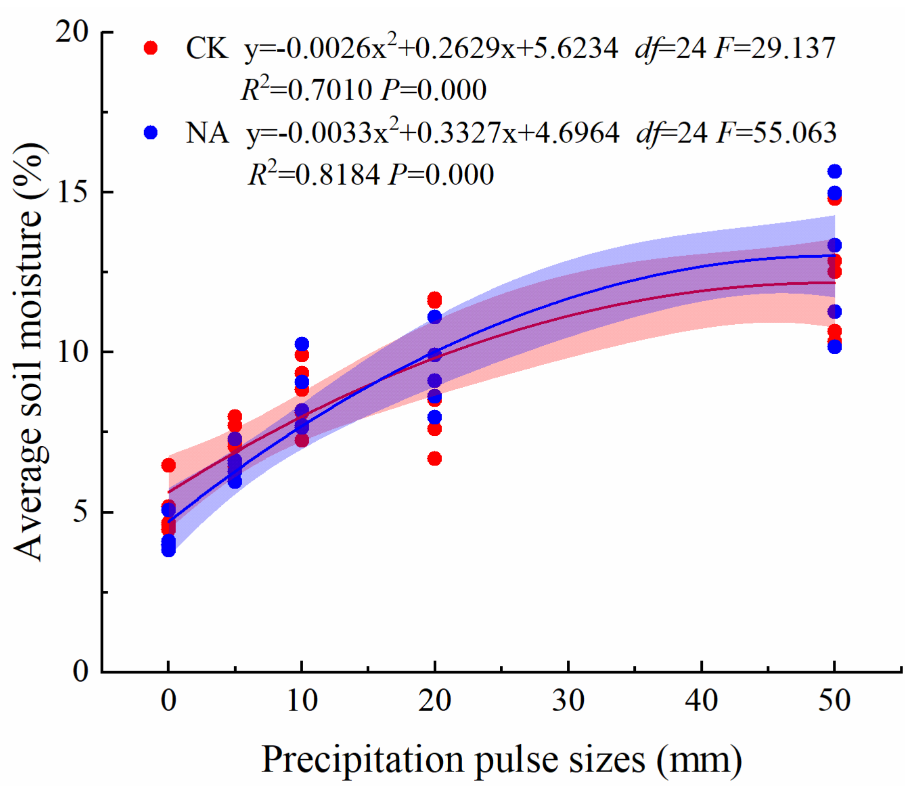

Supplementary Figure S1 The relationship between average soil moisture and precipitation pulse sizes after precipitation pulses and long-term N addition treatments.

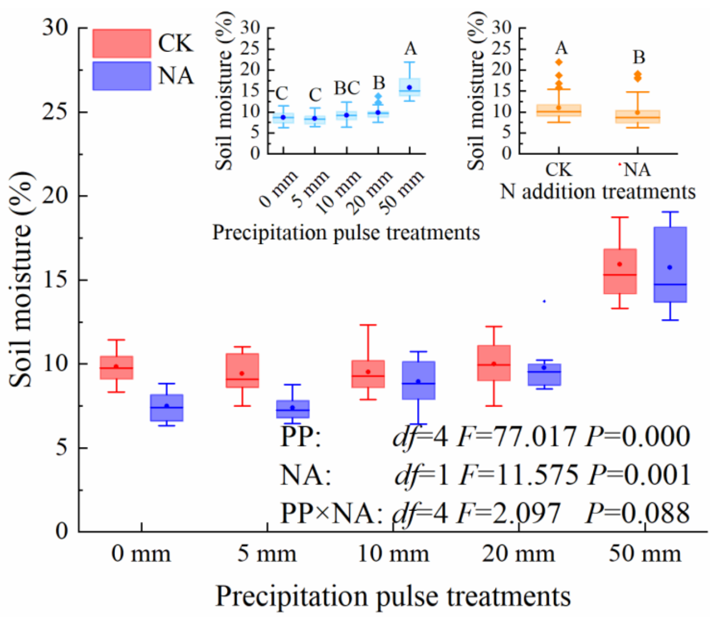

Supplementary Figure S2 Responses of average soil moisture at 10-30 cm depth to precipitation pulses and N addition treatments. The inserted graph (light blue column) shows the differences in the average soil moisture among the precipitation pulses. The inserted graph (orange column) shows the differences in average soil moisture between the control and N addition treatments. Boxplots show the median (lines within the box) and interquartile range (box boundaries). Whiskers extend to the most extreme data point within the 1.5 × (75-25%) data range. Different capital letters (above each boxplot) denote significant differences among the precipitation pulses or between the control and N addition treatments.

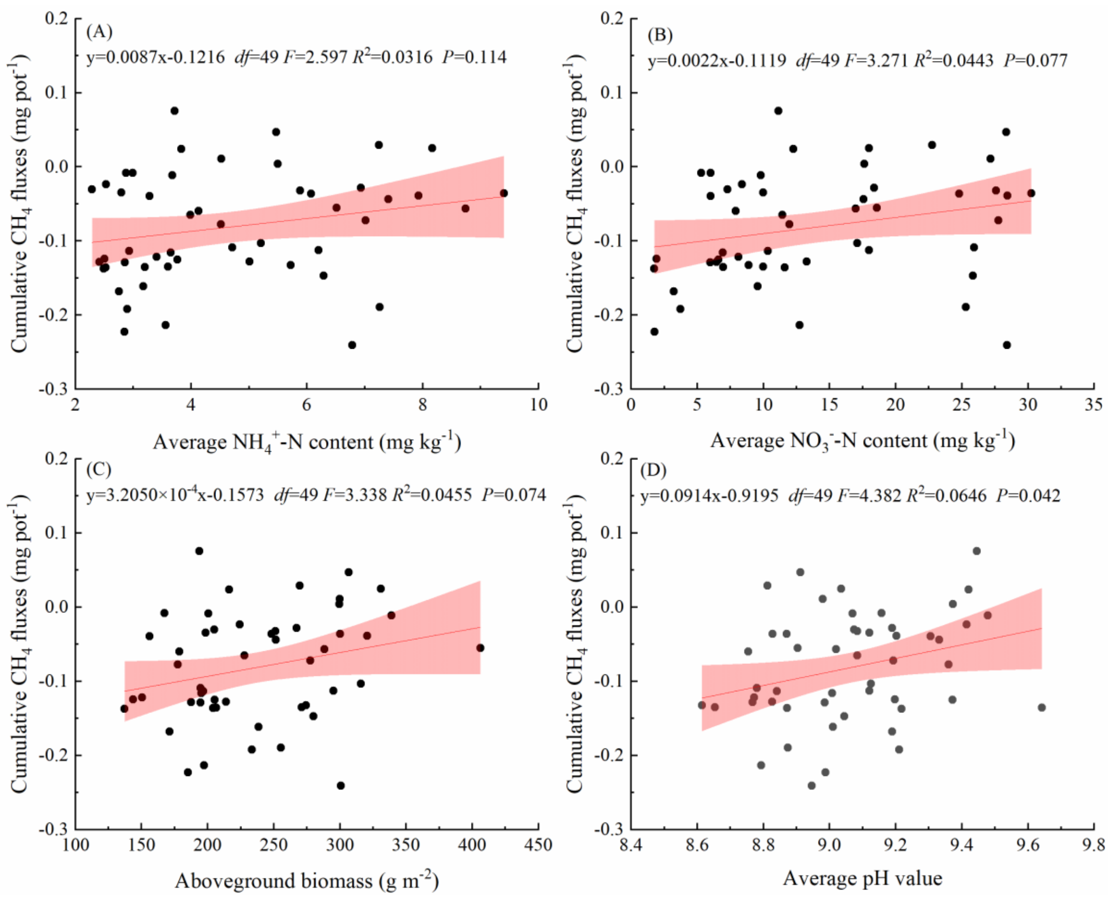

Supplementary Figure S3 Dependency of cumulative CH_4 fluxes on average NH_4^+-N content (A), average NO_3^--N content (B), aboveground biomass (C), and average pH value (D) after precipitation pulses and N addition treatments.

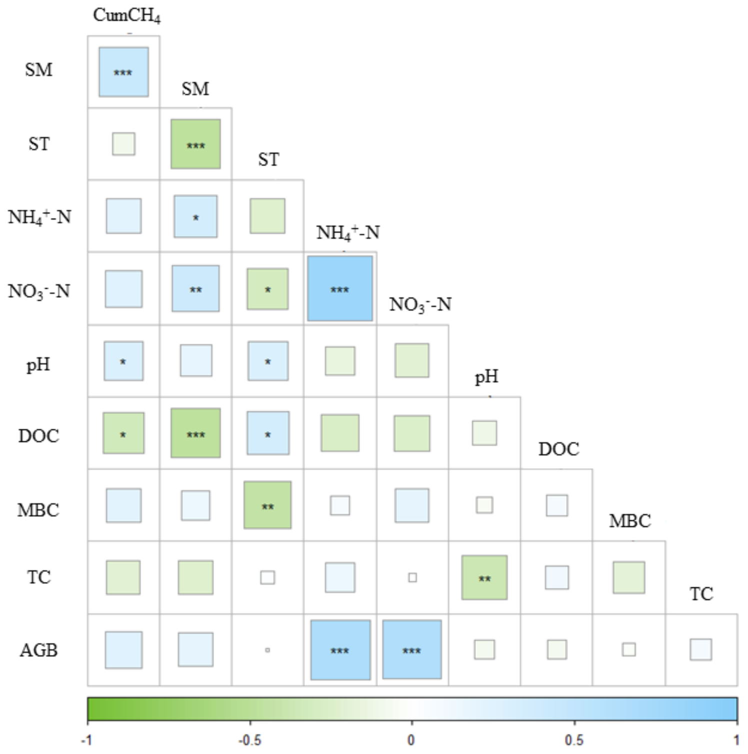

Supplementary Figure S4 The correlations between cumulative CH_4 emissions and biotic and abiotic factors after the precipitation pulses and long-term N addition treatments. CumCH4: cumulative CH_4 emissions; SM: soil moisture; ST: soil temperature; NH_4^+-N: NH_4^+-N content; NO_3^--N: NO_3^--N content; pH: pH value; DOC: dissolved organic carbon; MBC: microbial biomass carbon; TC: total carbon; AGB: aboveground biomass. *, $P < 0.05$; **, $P < 0.01$; ***, $P < 0.001$.
